# Pulsed Near Infrared Transcranial and Intranasal Photobiomodulation Significantly Modulates Neural Oscillations: a *pilot exploratory* study

**DOI:** 10.1038/s41598-019-42693-x

**Published:** 2019-04-19

**Authors:** Reza Zomorrodi, Genane Loheswaran, Abhiram Pushparaj, Lew Lim

**Affiliations:** 1 Temerty Centre for Therapeutic Brain Intervention, Centre for Addiction and Mental Health, Toronto, Ontario Canada; 2 Vielight Inc., Toronto, Ontario Canada; 3 Ironstone Product Development Inc. & Qunuba Sciences Inc., Toronto, Ontario Canada

**Keywords:** Neural circuits, Neural circuits, Neurophysiology, Neurophysiology

## Abstract

Transcranial photobiomodulation (tPBM) is the application of low levels of red or near-infrared (NIR) light to stimulate neural tissues. Here, we administer tPBM in the form of NIR light (810 nm wavelength) pulsed at 40 Hz to the default mode network (DMN), and examine its effects on human neural oscillations, in a randomized, sham-controlled, double-blinded trial. Using electroencephalography (EEG), we found that a single session of tPBM significantly increases the power of the higher oscillatory frequencies of alpha, beta and gamma and reduces the power of the slower frequencies of delta and theta in subjects in resting state. Furthermore, the analysis of network properties using inter-regional synchrony via weighted phase lag index (wPLI) and graph theory measures, indicate the effect of tPBM on the integration and segregation of brain networks. These changes were significantly different when compared to sham stimulation. Our preliminary findings demonstrate for the first time that tPBM can be used to non-invasively modulate neural oscillations, and encourage further confirmatory clinical investigations.

## Introduction

Photobiomodulation (PBM) refers to the application of low levels of red or near-infrared (NIR) light to either stimulate or inhibit biological cells and tissues involving photochemical mechanisms^1^. It was first discovered in 1967 when Endre Mester observed that low powered laser treatment promoted hair regrowth and wound healing in rats^2,3^. This inspired numerous investigations into the use of low level lasers and light emitting diodes (LEDs) for therapeutic purposes, collectively termed ‘low level light therapy’ (LLLT). In 2015, a global initiative was taken by researchers in this field to standardize the term to ‘photobiomodulation’.

Transcranial PBM (tPBM), targeting delivery of light energy to the brain, is associated with increased cerebral blood flow, oxygen availability and consumption, adenoside triphophosphate (ATP) production, and improved mitochondrial activity^4^. More recently, tPBM has demonstrated its value as a treatment for neurological^5–10^ and neurodegenerative conditions, including Alzheimer’s disease^11,12^.

Thus, tPBM is a form of non-invasive brain stimulation (NIBS). However, compared to the more established forms of NIBS, such as transcranial magnetic stimulation (TMS) and transcranial direct current stimulation (tDCS), the concept of the brain being responsive to light stimulation is unfamiliar to many. In recent years, research on the potential efficacy of tPBM has gained momentum^13^. Research on the effect of PBM on brain cell recovery has shown that, under laboratory conditions, damaged neurons can regrow their neurites with direct exposure to visible red low level lasers^14^. In an animal study, PBM has been found capable of promoting neurogenesis after ischemic stroke through the proliferation and differentiation of internal neuroprogenitor cells^15^.

The effect of PBM on mitochondrial function is the most well investigated mechanism of its potential therapeutic effects^4^. PBM has been demonstrated to increase the activity of complexes in the electron transport chain of mitochondria, including complexes I, II, III, IV and succinate dehydrogenase^16^. In particular, increased activity of the transmembrane protein complex IV, also known as the enzyme cytochrome c oxidase, during PBM results in increased ATP production^16^. Furthermore, PBM results in activation of signaling pathways and transcription factors resulting in increased expression of genes related to protein synthesis, cell migration and proliferation, anti-inflammatory signaling, anti-apoptotic protein and antioxidant enzymes^4^.

In a recent review of NIBS methods that included a comparison between tDCS, TMS and tPBM, Giordano *et al*., recognized that the mechanisms of tPBM are better understood than tDCS but “there is little evidence to date that (tPBM) produces direct neural activity”^17^. It is therefore timely that this study presents the effects of tPBM in terms of electrophysiological measures of the brain using electroencephalography (EEG). Apart from the fundamental mitochondrial-based mechanism resulting from the delivery of NIR to brain tissues, there have been indications that certain adjustable parameters may have an influence on neural activities, particularly the pulsing rate of the NIR delivery^12,18,19^. This double-blind, crossover study gives us the opportunity to analyze objective data to understand the effect of a selected pulse frequency of 40 Hz and other PBM parameters on neural activity. It may open up the possibility of exploring the effects of alternative PBM parameters in future studies.

## Methods

### Study Design

**Figure 1:**
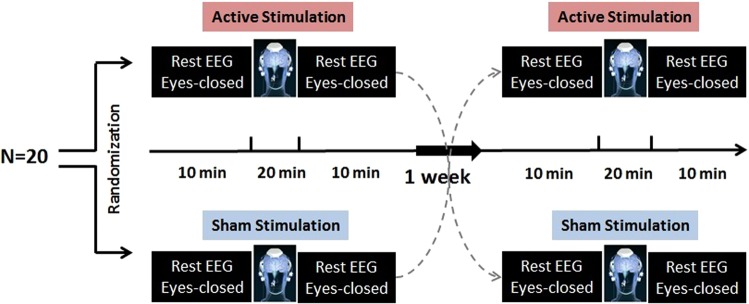
Schematic diagram of study design. Twenty healthy participants randomized to receive either active or sham tPBM with a minimum 1-week washout period between the two visits. 10 minutes eye-closed rest EEG recorded pre and post of each intervention.

This was a small, exploratory, double-blind, prospective cross-over study. Study subjects attended two study visits (Fig. 1): one visit where the subjects received active tPBM stimulation with rest EEG and another visit where the subjects received sham tPBM stimulation with rest EEG. The orders of the two visits were randomized and there was a minimum one-week washout period between the two visits.

### Study Visits

At the beginning of the first study visit, each subject was screened for eligibility before proceeding with enrollment. Then the subject was asked to be seated in a comfortable chair and a 10 minute eyes closed EEG recording was collected. Next, an active or sham tPBM device (described below) was positioned on the subject’s head and turned on for 20 minutes. Then the device was removed and another 10 minute eyes closed rest EEG was recorded.

### Subjects

**Figure 2:**
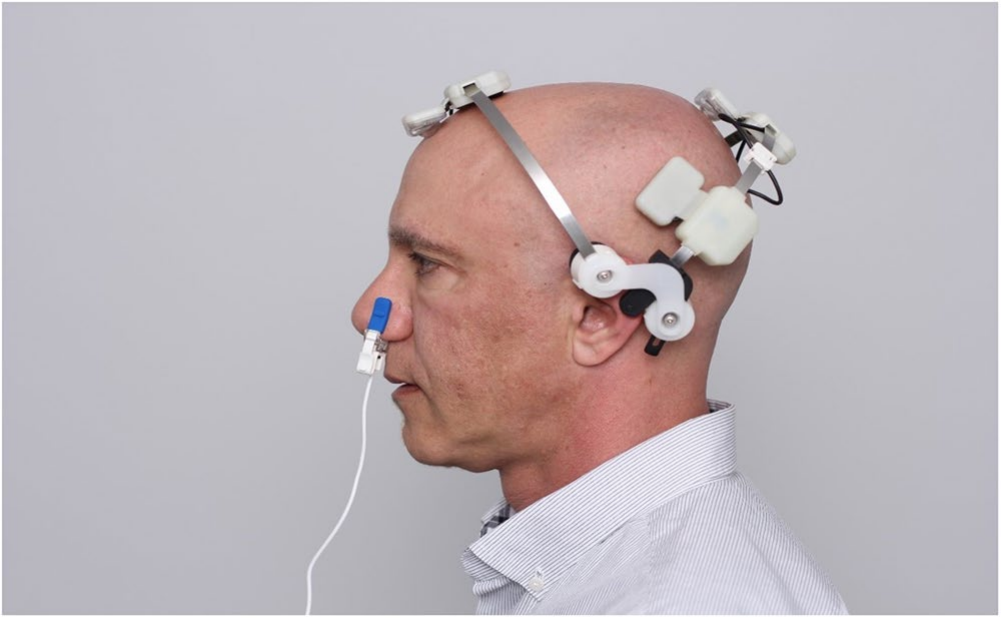
The Vielight Neuro Gamma in use. The stimulation modules consist of a Nasal Applicator, and a Head Set with four light emitting diode (LED) modules intended to be positioned over the hubs of the default mode network (DMN).

Twenty healthy adults (mean age 68.00 ± 5.94, 61–74 years of age, 9 Males) were recruited (Table 1). Written informed consent for participation in the study was obtained from all the subjects prior to enrollment into the study. The study was conducted in compliance with the Declaration of Helsinki and was approved by the research ethics board of IRB Services Canada. The individual included in Fig. 2 and Supplementary Fig. 2 has provided informed consent for publication of identifying information/images in an online open-access publication. All data was anonymized and no subject identifying information or images are published. Subjects were excluded if they had a Mini-Mental State Examination Score <27, a current major psychiatric or neurologic disease, a history of stroke, seizures and/or a medical condition uncontrolled with stable therapy. Subjects were compensated for their participation at the end of the last study visit.

**Table 1:** Demographic characteristics of the study population.

Gender	Sample Size (N)	Age (years)	Education (years)
Female	11	69 ± 6.32	12 ± 4.11
Male	9	66 ± 4.02	14 ± 3.71

### tPBM device

The tPBM device used in the study was the ‘Vielight Neuro Gamma’ (Neuro Gamma). It is a portable, wearable, low-level light delivery device that administers near-infrared light to the brain transcranially and intra-nasally. Its specifications and intended use fall within the definition of a low risk general wellness device in a policy guideline released by the Center for Devices and Radiological Health of the United States Food and Drugs Administration in 2016, which exempts it from medical device regulations^20^. The device consists of a controller, a nasal applicator, and a head set with four light emitting diode (LED) modules (Fig. 2).

The Neuro Gamma delivers painless, non-invasive, non-thermal, non-laser, pulsed (40 Hz; 50% duty cycle), near-infrared light (810 nm wavelength) through 5 non-laser LEDs over a 20- minute session. The device is powered by three rechargeable NiMH batteries. The LEDs have been designed to be positioned to deliver the near infrared light (NIR) to the subdivisions of the default mode network (DMN)^19^. These subdivisions include the ventral medial prefrontal cortex (vmPFC); the dorsal medial prefrontal cortex (dmPFC); the posterior cingulate cortex (PCC); adjacent precuneus (PCu) plus the lateral parietal cortex (LPC) and the entorhinal cortex (EC)^19^. “Hubs” and “nodes” described in literature are synonymous with these subdivisions or are located within the vicinity of these subdivisions. As shown in Fig. 2, one of the Neuro Gamma LEDs is placed inside the nose for intranasal transmission of NIR light to the ventral section of the brain which includes the vmPFC and the olfactory bulb which has a direct activating projection to the EC and parahippocampal area^21^. The remaining 4 LEDs on the headset are positioned over selected locations to direct NIR to the neocortical subdivisions of the DMN - the dmPFC, PCu, and posterior PCC (NIR light directed through the left and right angular gyri). These locations correspond to FPz, Cz, T3 and T4 respectively, described under the 10–20 EEG montage.

The group of LEDs containing those positioned on the dmPFC and intranasally (Group A LEDs) pulse in synchrony (in-phased) and the other group of LEDs on the PCu and left and right LPCs (Group 2 LEDs) also pulse in synchrony (in-phased) within its group. Between Group A and Group B LEDs, the pulsing frequencies are completely asynchronous (out-of-phase).The power density output of the nasal applicator is 25 mW/cm^2^, anterior LED is 75 mW/cm^2^, and three posterior LEDs, 100 mW/cm^2^. Over the set-time of 20 minutes, the energy dose to the brain (headset and intranasal applicator) equals to 240 J/cm^2^. Detailed specifications and parameters are set out in Table 2.

**Table 2:** Vielight Neuro Gamma Parameters.

Source	LED
Wavelength (nm)	810
Power output of LED on the anterior band (mW)	75(transcranial)
Power output of each LED on the posterior band (mW)	25(intranasal)
Power density of LED on the anterior band (mW/cm^2^)	100(transcranial)
Power density of each LED on the posterior band (mW/cm^2^)	25(intranasal)
Pulse frequency (Hz)	40
Pulse duty cycle, percentage	50
Duration of each treatment session (min)	20
Beam spot size (cm^2^)	≈1
Total energy delivered per session (Joules)	240
Total energy density per session (Joules/cm^2^)	240

The Neuro Gamma has been independently tested by TUV SUD Canada for Electrical Safety as well as Emissions & Immunity for Multimedia Class B Equipment.

### EEG acquisition

The EEG signals were recorded using the DISCOVERY 24E (Brainmaster Inc.) at 256 Hz sampling rate and a bandwidth of 0.43–80 Hz. The amplifier was a low-noise DC-sensitive and a 24-bit analog-to-digital device. For the EEG cap, we used a 19-Channel free-cap set (Institut für EEG-Neurofeedback), which allows EEG to be recorded during tPBM delivery. The EEG channels located at a 10–20 montage on the elastic net. The data was collected during the 10-minute sessions with the subjects at rest and with the eyes closed, before and after Neuro Gamma stimulation.

### EEG data preprocessing

EEG data was processed offline using a custom MATLAB script (MathWorks, MA, USA), and EEGLAB toolbox (Swartz Center for Computational Neuroscience, University of California at San Diego) in the following sequence. First, EEG data were visually inspected to remove noisy channels or highly contaminated artifacts. Thereafter, EEG data were digitally filtered by using a second order, Butterworth, zero-phase shift 1–55 Hz band pass filter (24 dB/Oct), and then segmented into 2 sec epochs. Then, an electrodes-by-trials matrix of ones was created and assigned a value of zero if an epoch had: (1) amplitude larger than +/−150 μV; (2) standard deviation 3 times larger than the average of all trials; or (3) power spectrums that violated 1/f power law. An electrode was rejected if its corresponding row had more than 60% of columns (trials) coded as zeros. An epoch was removed if its corresponding column had more than 20% of rows (electrodes) coded as zeros. An independent component analysis (ICA) (EEGLAB toolbox; Infomax algorithm) was performed to remove ocular, muscle artifacts, and other noise from the EEG data. Finally, the data was re-referenced to the average for further analysis.

### EEG analysis of power, network connectivity and synchrony

The power spectrum analysis was conducted using the spectopo () function as implemented in EEGLAB, using Welch’s method (with a window length of 512 points, (fft) length of 1024 points, non-overlap). The absolute power was calculated for each channel and each frequency band: Delta (1–3) Hz, Theta (4–7) Hz, Alpha (8–14) Hz, Beta (14–30) Hz and Gamma (30–50) Hz.

To explore a possible change in brain functional connectivity and synchrony, we used the weighted phase lag index (wPLI) and graph theory measure. The weighted phase lag index is a functional connectivity measure and assesses the phase ‘lagging’ consistency between each pair of EEG channels. The advantages of wPLI over other cerebral synchrony assessments are its lower sensitivity to noise and volume conduction effects, therefore providing more reliable evaluation of long range synchronization of neural activity^22^. The wPLI ranges between 0 (no phase consistency) and 1 (full synchrony).

A network based on wPLI was constructed and graph measures were employed to evaluate network integration, segregation and quantifying efficiency of information transfer^23,24^. Four graph measures were computed using the Brain connectivity toolbox (BCT)^25^: (I) The characteristic path length (CPL), which is the average length of all pairwise shortest paths connecting any node to another, to assess integration; (II) the clustering coefficient (CC), which is the mean nodal CC averaged across all vertices to assess segregation; (III) the global network efficiency, which quantifies the exchange of information across the whole network; and (IV) the local network efficiency, which quantifies a network’s resistance to failure on a small scale and characterizes how well information is exchanged by its neighbors when a node is removed.

EEG channels and the value of wPLI represent nodes (i.e, 19) and edge (i.e., 19 × 19) of the network, respectively. For each frequency band, we applied different sparsity thresholds by choosing 5–100% of the strongest wPLI to binarize the edges. The sparsity-based threshold ensures the same number of edges for each network. Then, we evaluated the significance of changes in the network properties before and after active and sham stimulations for different sparsity levels.

### Statistical analyses

Differences in power spectrum over all electrodes were statistically assessed using a cluster-based permutation test that identifies clusters of electrodes with significant changes, while correcting for multiple comparisons^26^. Cluster-level statistics were computed by taking the sum of the t-values over adjacent neighboring electrodes. Clusters were defined as two or more spatially contiguous electrodes in which the t-statistics of power spectrum exceeded a chosen threshold of alpha level of p < 0.05 and α_cluster_ = 0.01. The null distribution was obtained by randomly permuting 1000 pieces of data (i.e., randomizing data across pre and post tPBM and rerunning the statistical test). The Matlab toolbox Fieldtrip was used for this analysis^13,27^. To summarize the data, we averaged the power of 19 EEG electrodes for each subject and ran two sided nonparametric Wilcoxon tests to compare the ratio of post over pre-stimulation values of active and sham conditions.

At each level of network sparsity level, the brain network properties were compared using two sided nonparametric Wilcoxon paired-sample t-tests. The brain networks with different sparsity levels are considered independent graphs, and thus the correction for multiple comparisons (e.g. Bonferroni correction) is not required^24,25^.

## Results

### Power spectrum analysis

**Figure 3:**
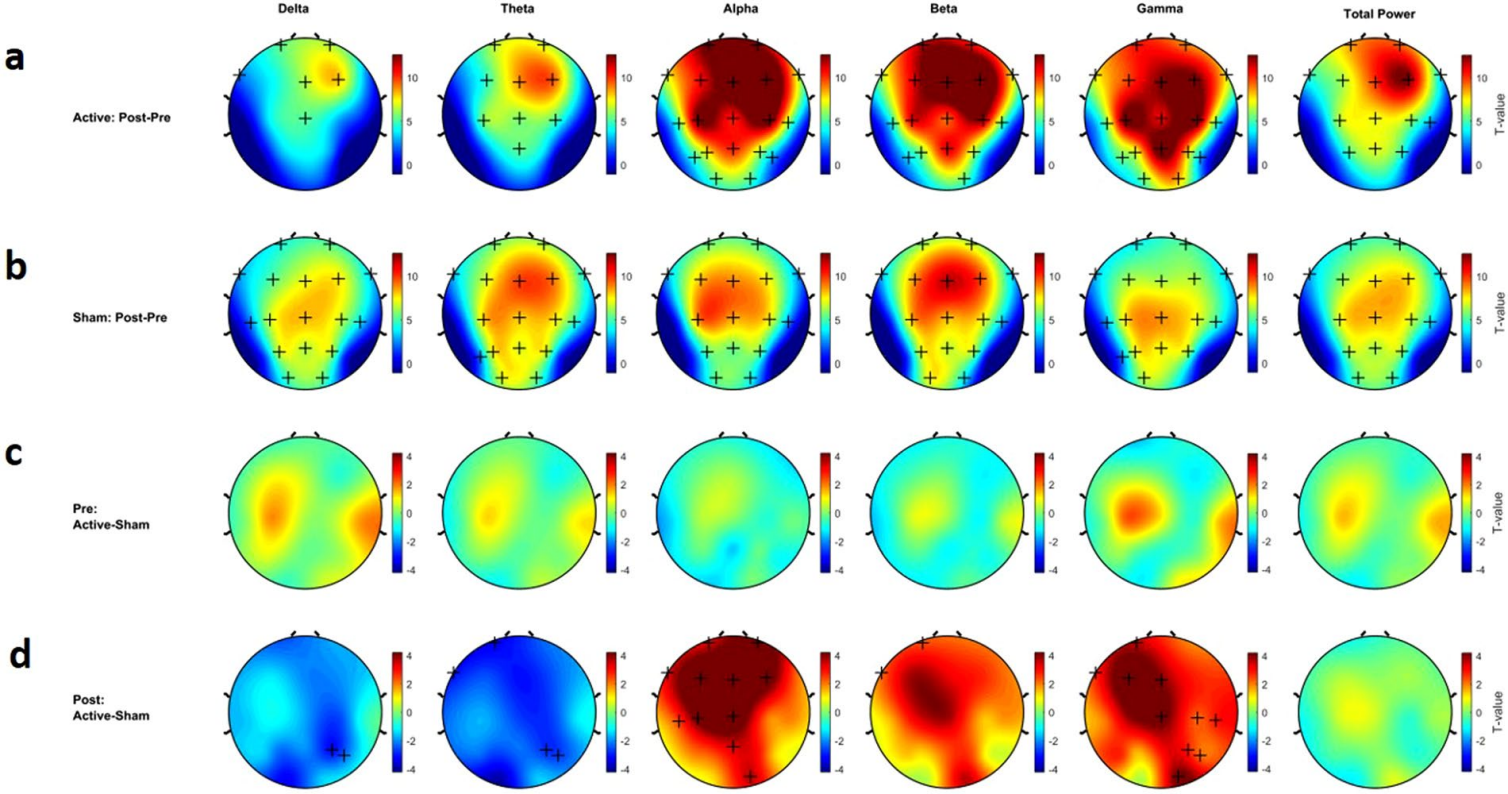
Non-parametric cluster-based permutation test comparing the rest EEG power spectrum between active and sham tPBM. Topographical maps are color-coded according to the permutation tests t-values. Clusters of electrodes with significant difference between the two conditions are marked in ‘+’ sign (p < 0.05 and α_cluster_ = 0.01). (**a**) Difference between post and pre active tPBM. (**b**) Difference between post and pre sham tPBM. (**c**) Difference between pre active and sham tPMB. (**d**) Difference between post active and sham tPMB.

Cluster-based permutation tests, with t-values presented by topological maps, are reported in Fig. 3 for comparison between pre- and post-tPBM sessions in both sham and active conditions. In Fig. 3, clusters of electrodes with power values that are significantly different (p < 0.05 after Bonferroni correction) between the two conditions are marked by a plus sign. Decreases or increases in absolute power are color coded in dark blue and dark red, respectively.

Significant absolute power spectrum alterations after 20 minutes of active tPBM were observed in all oscillatory frequency bands. Figure 3a illustrates the pre- and post-stimulation differences in power spectrum for active tPBM. In the delta and theta frequency bands, there were significant increases in power in the centro-frontal electrodes (4 < t-values < 6, p < 0.05) but no significant difference in the temporal, parietal and occipital electrodes (t = 0.156, p > 0.05). In the alpha, beta and gamma bands, we observed significant increases (t-value > 7, p < 0.05) in almost all of the electrodes. Twenty minutes of sham tPBM resulted in significant changes in power spectrum, but with non-specific locations and frequency signatures (5 < t-values < 6, p < 0.05) (Fig. 3b). The comparison between rest-EEG before (or pre) both active and sham conditions (1 week interval) showed no significant difference across all electrodes (Fig. 3c). However, the comparison between rest-EEG after (or post-) active and sham conditions revealed a distinct effect of active tPBM on the power spectrum. Our data showed: (I) decreases in the lower oscillatory frequencies (i.e., delta and theta) with significant reductions in the posterior region and, (II) increases in higher oscillatory frequency bands with significant alterations in centro-frontal regions for alpha and beta, and a significant global change in gamma power (t-value > 3, p < 0.05) (Fig. 3d).

**Figure 4:**
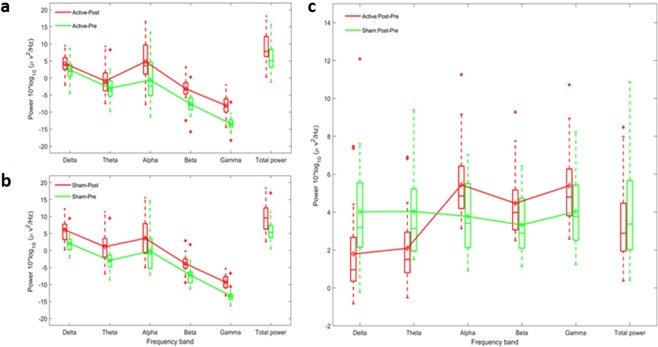
Influence of tPBM on resting-state electroencephalography. Box plot illustrates the median and range of power spectrum across all electrodes for each oscillatory frequency bands. (**a**) Effect of active tPBM on power spectrum pre (green line) and post (red line). (**b**) Effect of sham tPBM on power spectrum pre (green line) and post (red line). (**c**) Difference between Active and Sham tPBM: Change of power spectrum Post-Pre for active (red line) and sham (green line) tPBM. Active versus sham stimulation revealed significant lower alteration in delta and theta power and higher change in alpha, beta and gamma frequency bands.

To evaluate the overall tPBM effects, we averaged the power spectrum across all electrodes for each oscillatory frequency band and compared the ratio of post- over pre-session rest-EEG for both active and sham conditions. Figure 4 illustrates the boxplot of absolute power ratio. There was an overall increase in power when comparing post- to pre-stimulation in both the active (t = 6.855; p < 0.01; Fig. 4a) and sham stimulation conditions (t = 6.996; p < 0.01; Fig. 4b). This increase was seen in all frequency bands for both sham (delta: t = 3.403, p < 0.01; theta: t = 4.415, p < 0.01; alpha: t = 11.876, p < 0.01; beta: t = 10.756, p < 0.01; gamma: t = 10.876; p < 0.01; Fig. 4b) and active stimulation (delta: t = 6.177, p < 0.01; theta: t = 7.589, p < 0.01; alpha: t = 8.572, p < 0.01; beta: t = 9.553, p < 0.01; gamma: t = 8.637; p < 0.01; Fig. 4a). Interestingly, the change in power was frequency dependent during active stimulation. In the active stimulation, there was a suppression in the increase in power of the delta and theta bands that was observed during sham stimulation. Conversely, active stimulation produced a facilitation of the increase in power in alpha, beta and gamma compared to sham.

A comparison between the active and sham groups presented significant differences in delta (t = −3.513 p < 0.01), theta (t = −3.736 p < 0.01), alpha (t = 4.455 p < 0.01), beta (t = 3.221 p < 0.01), and gamma (t = 2.658, p < 00.1) frequency bands (Fig. 4c).

### Brain network functional connectivity and synchrony analyses

**Figure 5:**
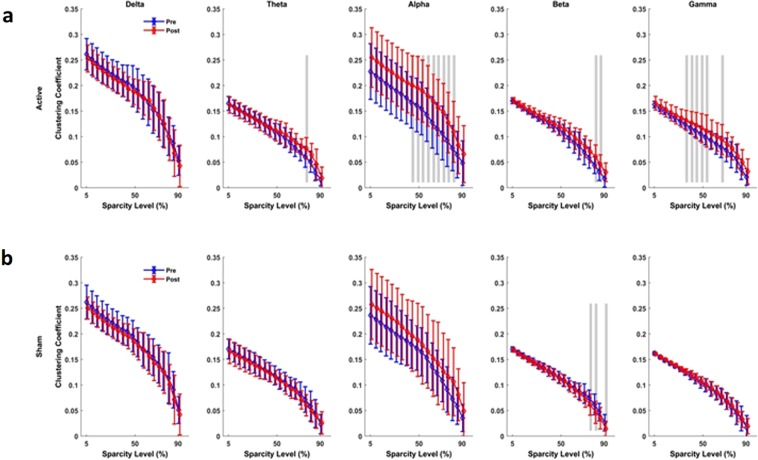
Connectivity assessment using Clustering coefficient (CC). (**a**) Active tPBM caused index significantly changed CC for wide range of 45–80% sparsity levels in the alpha band, and 35–55% sparsity levels in the gamma band. (**b**) Sham tPBM did not caused significant change in CC index, but beta at 75, 82 and 90% of sparsity levels. Blue and red lines line indicate pre and post conditions, respectively. The gray lines indicate a significant difference (p < 0.01) at a certain sparsity level.

**Figure 6:**
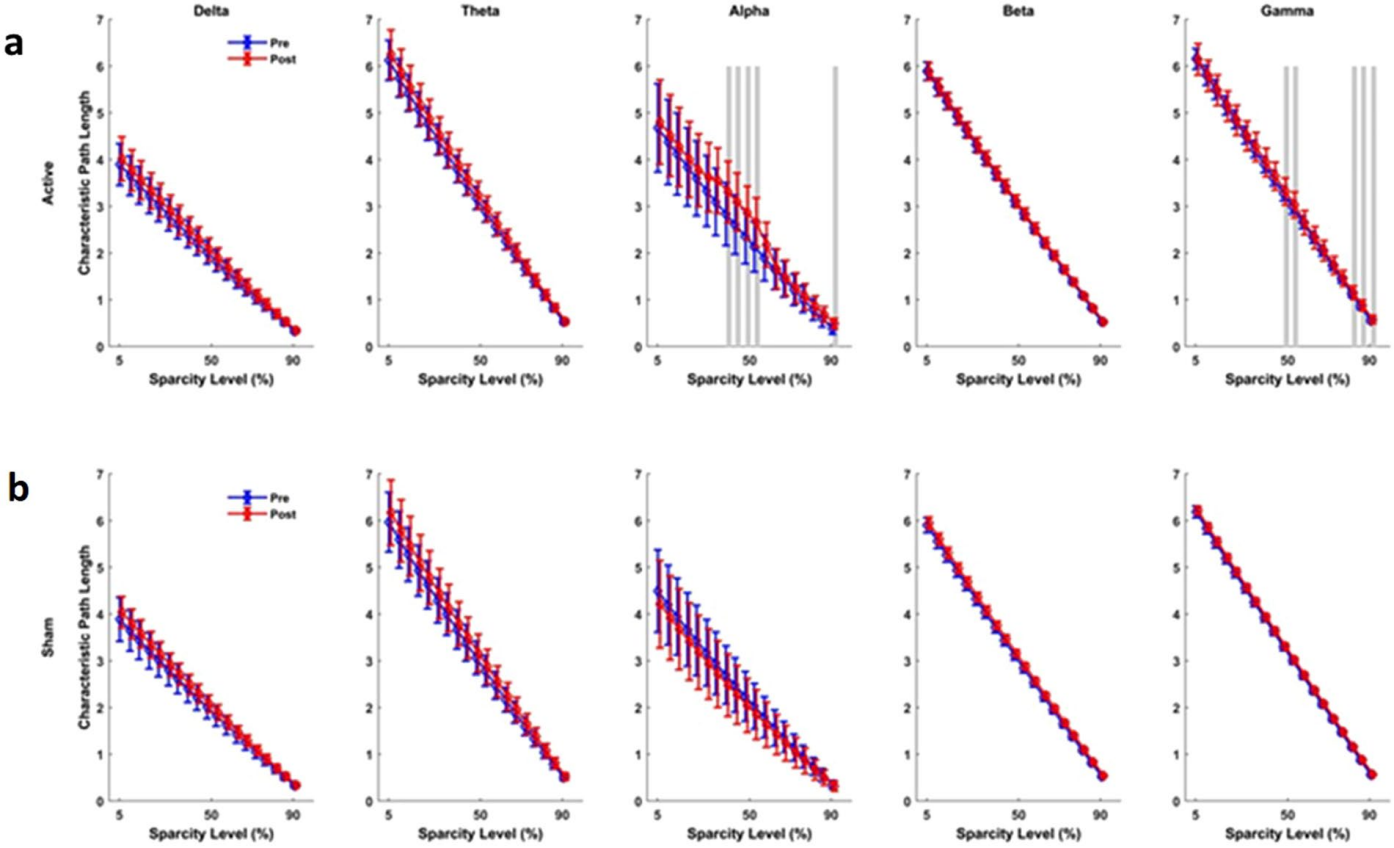
Connectivity assessment using the characteristic path length (CPL). (**a**) Active tPBM caused index significantly changed CLP in the alpha band for 40–55% of sparsity levels and the gamma band for 50–55% and 80–85%. (**b**) Sham tPBM did not cause any significant change in CPL index. Blue and red lines line indicates pre and post conditions, respectively. The gray lines indicate a significant difference (P < 0.01) at a certain sparsity level.

**Figure 7:**
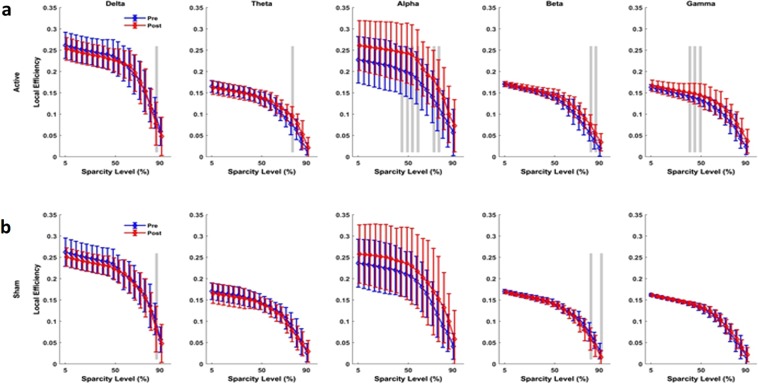
Connectivity assessment using Local Efficiency measure. (**a**) Active tPBM caused significant changes in network local efficacy mostly in the alpha band for 45–60% and 70–75% of sparsity levels, in the gamma band for 40–50% of sparsity levels, in the beta band for 80–85% of sparsity levels, and in delta and theta bands for 85% and 80% of sparsity levels, respectively. (**b**) Sham tPBM did not cause any significant change in the local efficacy except in the beta band for 80 and 90% of sparsity levels. Blue and red lines line indicate pre and post conditions, respectively. The gray lines indicate a significant difference (P < 0.01) at a certain sparsity level.

**Figure 8:**
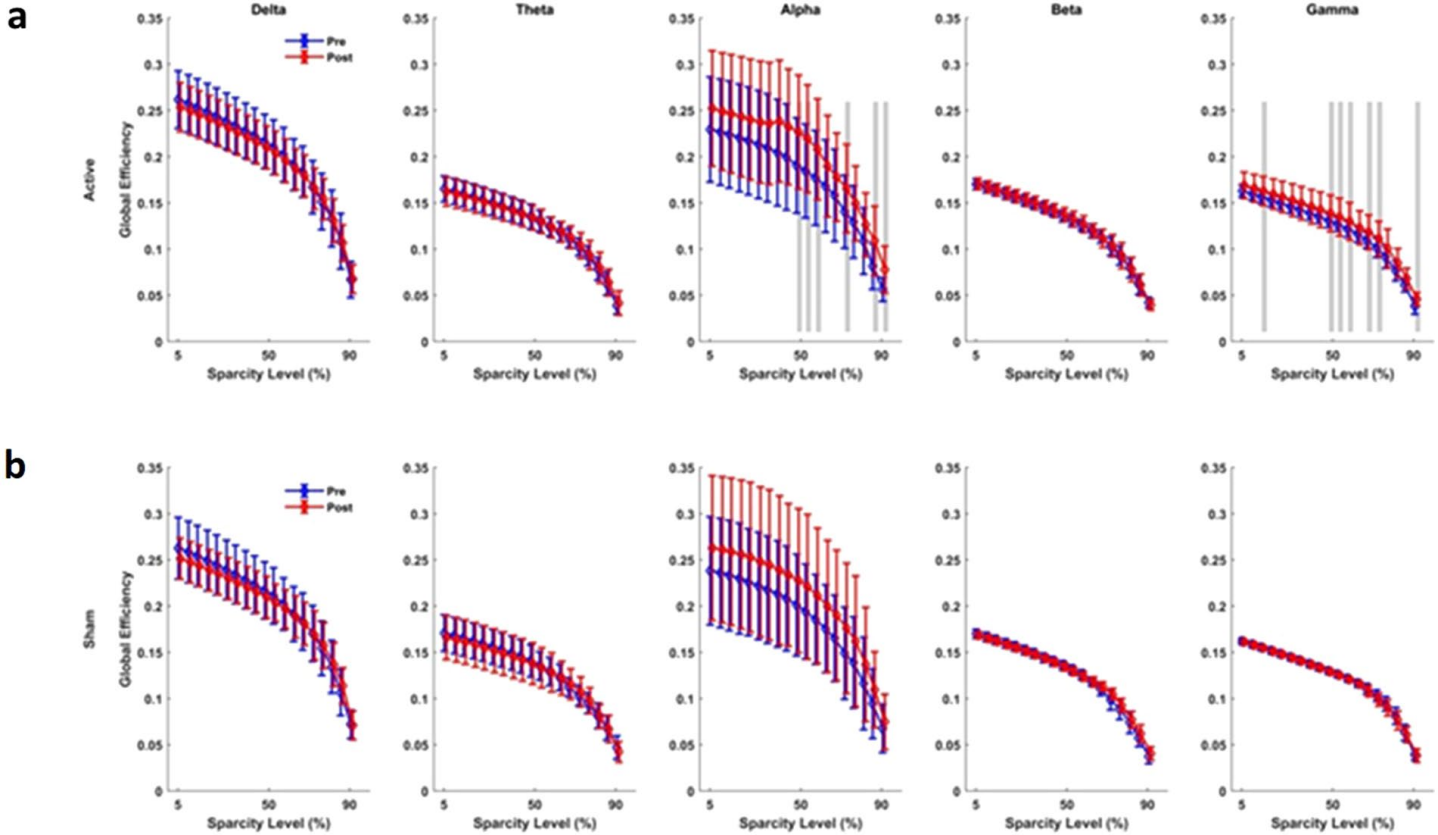
Connectivity assessment using Global Efficiency measure. (**a**) Active tPBM caused significant changes in network global efficacy in the alpha band for 50–60%, 75%, 85–90% of sparsity levels, in the gamma band for 15%, 50–60%, 70–75% and 90% of sparsity levels. (**b**) Sham tPBM did not cause any significant change in the global efficacy. Blue and red lines line indicate pre and post conditions, respectively. The gray lines indicate a significant difference (P < 0.01) at a certain sparsity level.

Brain network connectivity and synchrony analyses in this study were based on the weighted phase lag index (wPLI) and graph measures to show changes in the clustering coefficient, characteristic path length (CPL) and local efficiency measures. For each frequency band and each sparsity level, mean and standard deviation of network indexes were plotted for both active and sham tPBM conditions (Figs 5–8).

The results of the functional network connectivity and synchrony analyses for different measures are as follows: For the active device, (I) The average cluster coefficient (CC) showed significant changes for a wide range of sparsity levels: 45–80% sparsity levels in the alpha band, 35–55% and 65% sparsity levels in the gamma band, 75% in the theta band, and 80–85% in the beta band (Fig. 5a). When the sham device was used (Fig. 5b), the average CC did not show significant changes for most of the sparsity levels and frequency bands, apart from the 75%, 82% and 90% sparsity levels in the beta band. (II) The characteristic path length (CPL) showed a significant change only for active tPBM in the alpha band for 40–55% sparsity levels and in the gamma band for 50–55% and 80–85% sparsity levels (Fig. 6a). Sham treatments did not cause changes in the CPL for sparsity levels in any frequency band (Fig. 6b). (III) After active tPBM, local efficiency of the network changed mostly in the alpha band for 45–60% and 70–75% sparsity levels, in the gamma band for 40–50% sparsity levels, in the beta band for 80–85% sparsity levels, and in delta and theta bands for 85% and 80% sparsity levels, respectively (Fig. 7a). In the sham treatment condition, data showed alterations in local efficiency in the beta band for 80% and 90% sparsity levels and in the delta band at 80% (Fig. 7b). (IV) After active tPBM treatments, the global efficiency of the network changed mostly in the alpha band for 50–60%, 75%, 85–90% sparsity levels, and in the gamma band for 15%, 50–60%, 70–75% and 90% sparsity levels (Fig. 8a). The global efficiency in sham condition did not change in any frequency band (Fig. 8b).

### Absence of Side Effects

Throughout the study, none of the 20 participants self-reported any adverse events or any unusual sensation.

## Discussion

The ease-of-use of tPBM, along with its seemingly advantageous tolerability profile, make it an appealing non-invasive brain stimulation (NIBS) method. To date, no published study has demonstrated the effect of tPBM on neural activities and brain oscillations. In this cross-over, double-blind study, our results revealed a significant effect of transcranial near-infrared light (810 nm wavelength) at 40 Hz pulsing rate on the power, functional connectivity and synchrony of endogenous brain activity.

Both the active and sham stimulations produced an increase in power in each of the frequency bands compared to baseline. This increase in power in sham stimulation was unexpected and suggests that resting wakefulness increases cortical activation. Previous studies have demonstrated that subjective sleepiness following prolonged periods of wakefulness (ie. 40 hours) have resulted in differential changes in the power of various frequency bands during the resting wakefulness state, measured with EEG^28,29^. However, to our knowledge, there has been no report on the effects of 30 minutes of resting state on the power density spectrum in absolute values.

While a consistent increase in power was observed across all the frequency bands with both active and sham stimulations, the change in power was frequency dependent with active stimulation. Compared to sham, active stimulation presented suppression of the increase in the lower frequency bands (delta and theta) and a further increase in power in the higher frequency bands (alpha, beta, and gamma). Interestingly, higher power in the lower frequency bands and reduced power in the higher frequency bands have been associated with disorders involving cognitive impairment, such as dementia and Alzheimer’s disease^30–34^. The data observed from the use of the active tPBM device has produced relevant results that are counter to the power spectrum characteristics of those conditions. Therefore, this suggests that active stimulation with 40 Hz NIR tPBM might be a desirable intervention to improve cognitive impairment, and could potentially improve the cognitive function of patients with dementia and Alzheimer’s disease^35,36^.

The significant alteration in the power spectrum and functional network properties in the alpha frequency band could be due to the targeting of the default mode network (DMN) through its recognized hubs/subdivisions emphasized by EEG recording of subjects in resting wakefulness^37–40^. The DMN is a network of brain regions that are activated when the mind wanders without engaging in tasks such as attention or action, and it is associated with introspection^37,41^, which are also largely characteristic of the brain in alpha state. Among all DMN hubs, the medial prefrontal cortex (mPFC) plays a mechanistic role in the alpha generation process^37,42^. Increased alpha power is posited to aid in the inhibition of irrelevant cortical areas while integrating relevant ones, sharing another introspective characteristic in the function of the DMN^43–45^. Additionally, spontaneous self-referential thought is linked to the increase of alpha power in the posterior DMN (including the precuneus and posterior cingulate) and gamma oscillations in the mPFC^38,41,46,47^. Several studies demonstrate deactivation of DMN during task-related activities and its essential role in alternating between internal and external attention^48^. Pathologies affecting the DMN include dementia, schizophrenia, autism, anxiety and depression^42,49^, suggesting the importance of normalizing DMN functions. A way to achieve this could be through normalizing alpha frequency oscillation.

Additionally, by analyzing brain network properties using wPLI and the graph theory measures, we observed significant effects of active tPBM. The connectivity measures, which assessed the integration and segregation properties of the network, showed a significance increase in clustering coefficient, characteristic path length (CPL) and local efficiency measures for each oscillation frequency band. The changes were most prominently in the alpha frequency bands. The increased connectivity and synchrony achieved by inducing 40 Hz to the DMN is novel. It warrants new investigations on how this could help with conditions that have characteristically low connectivity and synchrony in fast frequency bands.

A recent study by Chao and colleagues also demonstrated the impact of tPBM on DMN connectivity, specifically increased connectivity in the posterior cingulate cortex and lateral parietal lobes, using functional magnetic resonance imaging (fMRI). This increase in connectivity was correlated with improved cognition in patients with dementia^50^.

The pulse frequency employed likely plays an important role in the effects of tPBM on brain activity. Pulsing NIR light not only minimizes the heating effect and increases the possible penetration depth^51,52^, but may effectively interact with cellular activity via two proposed mechanisms by: (a) impacting the ionic channels kinetic such as potassium and calcium in the mitochondria^16,51,53^ (b) increasing the dissociation rate of nitric oxide from cyctochrome c oxidase^9,16,54^. A study by Iaccarino *et al*. has demonstrated that the visual processing of 40 Hz pulsing light significantly reduces β-amyloid deposits in the visual cortex of an AD animal model, similar to that observed from optogenetic “gamma entrainment” of fast-spiking parvalbumin-positive interneurons^19^. This finding further suggests that the 40 Hz pulsing of NIR tPBM should be further explored to address AD pathology.

We hypothesize that the action of 40 Hz NIR tPBM results in increased organization of neural function, which is likely to be accompanied by an increase in inhibition. During cognitive processes such as memory consolidation, the presence of gamma oscillations prevents neurotoxicity^55^. The amplitude of gamma oscillations is associated with GABA levels, with increased levels of GABA being correlated to an increased amplitude of the gamma band^56^. Future studies are required to confirm the effect of 40 Hz NIR tPBM on GABA levels.

There are several established theories for the ability of PBM to act locally and systemically; however, to our knowledge, this is the first study demonstrating an effect at the network level, through the observed changes in brain oscillations. This network effect may be mediated by increased intracellular Ca^2+^ concentration which has been observed at certain PBM doses^57,58^ potentially occurring through the engagement of the voltage-gated calcium channels^59^. Animal experiments have produced evidence of significant increases in the membrane potential with Ca^2+^ potentially underlying gamma oscillations of around 40 Hz^60,61^.

Together, these findings present the potential of tPBM as a valuable form of NIBS, offering a safe experimental tool to interact with the brain. PBM has minimal reported adverse effects, provided the parameters are understood at least at a basic level^62^. The potential of a tPBM device to significantly modulate the brain opens new opportunities for its use in research and therapeutic settings. This study presents for the first time, the significant modulatory effect tPBM has on the brain oscillatory patterns as measured with EEG. The main modulating parameters in the device used in the study are likely to be the 810 nm wavelength and the pulse rate of 40 Hz. Delivering these parameters to the hubs/subdivisions of the default mode network this way, significantly increased the power of the high oscillatory frequencies of alpha, beta and gamma; and reduced the power of the slower frequencies of delta and theta. These have been achieved after each single session of active treatment with the subjects in resting wakefulness state. In addition, the brain presented global inhibition, indicating increased organization.

Some important indications have been achieved in this study: (I) Despite not being obvious and no experience of sensation by the subject, NIR light delivered with relatively low power density can draw response from the brain that are measurable with EEG. (II) The significant effects of tPBM when applied as presented in this study. These promising preliminary results present a considerable forward step leading to the need of a larger confirmatory clinical investigation, which could establish the role of tPBM as an effective non-invasive neuromodulatory tool.

The potential capacity of tPBM to induce brain wave entrainment provides the opportunity to explore the effect of varying selected parameters, such as the pulse frequencies, location of the LED modules, power output, and pulse coherency between selected LEDs. For example, we could investigate the effect of two selected LED locations to pulse in-phase (to increase coherency/synchrony) or out-of-phase (to reduce coherency/synchrony). We could also investigate the differences in outcomes of pulse synchrony between brain networks or compare them with whole-brain synchrony. Answers to these investigations could potentially provide customized approaches to the modulation of brain wave patterns. The relatively quick brain response suggests that tPBM is a good candidate for use with neurofeedback methodologies, in order to optimize parameters for specific conditions. Medical conditions such as Alzheimer’s disease which express low power and synchrony in the alpha, beta and gamma and high power and synchrony in delta and theta brainwaves are obvious targets of future tPBM research. The adjustability of the various parameters of tPBM, along with the relatively quick response observed by EEG, is optimal for the exploration of customized treatments for varying indications.

## Supplementary information

Pulsed Near Infrared Transcranial and Intranasal Photobiomodulation Significantly Modulates Neural Oscillations: a pilot exploratory study
