# Supplementary material for: Pulsed Near Infrared Transcranial and Intranasal Photobiomodulation Significantly Modulates Neural Oscillations: a pilot exploratory study
Source: Sci Rep. 2019 Apr 19;9:6309. doi: 10.1038/s41598-019-42693-x (PMC6474892; doi:10.1038/s41598-019-42693-x)
Supplement: Supplementary file 1 — Pulsed Near Infrared Transcranial and Intranasal Photobiomodulation Significantly Modulates Neural Oscillations: a pilot exploratory study [file 41598_2019_42693_MOESM1_ESM.docx]

**Pulsed Near Infrared Transcranial and Intranasal Photobiomodulation Significantly Modulates Neural Oscillations:** **a pilot exploratory study**

| **Principal Investigator:**  Reza Zomorrodi, PhD  Project Scientist,  Temerty Centre for Therapeutic Brain Intervention  Centre for Addiction and Mental Health  Unit 4-1, Office 125A  1001 Queen Street West, Toronto, ON. M6J 1H4  Reza.Zomorrodi@camh.ca  (Tel) 416-535-8501 ext 30205 |
| --- |

Reza Zomorrodi^1,2^ PhD, Genane Loheswaran^2^ PhD, Abhiram Pushparaj^3^ PhD, Lew Lim^2^ PhD, MBA

^1^ Temerty Centre for Therapeutic Brain Intervention, Centre for Addiction and Mental Health; Toronto Ontario, Canada

^2^ Vielight Inc., Toronto, Ontario, Canada

^3^ Ironstone Product Development Inc., Toronto, Ontario, Canada

**Table of Contents:**

[1. STUDY SYNOPSIS 3](#_Toc527710101)

[2. INTRODUCTION 4](#_Toc527710102)

[2.1. Study Rationale & Background 5](#_Toc527710103)

[2.2. Study Device: Vielight Neuro Gamma 5](#_Toc527710104)

[2.3. Study Objective 6](#_Toc527710105)

[2.1. Study Design 6](#_Toc527710106)

[2.2. Study Endpoints 6](#_Toc527710107)

[2.3. Study Site 6](#_Toc527710108)

[3. SUBJECT ELIGIBILITY 6](#_Toc527710109)

[3.1. Inclusion Criteria 6](#_Toc527710110)

[3.2. Exclusion Criteria 7](#_Toc527710111)

[4. STUDY EXECUTION 7](#_Toc527710112)

[4.1. Subject Recruitment 7](#_Toc527710113)

[4.2. Preliminary Eligibility Assessment 7](#_Toc527710114)

[4.3. Study Visit Procedures 7](#_Toc527710115)

[5. STUDY VISIT EQUIPMENT 9](#_Toc527710116)

[5.1. Devices and Procedural Purposes 9](#_Toc527710117)

[5.2. Device Safety 9](#_Toc527710118)

[6. STATISTICAL PLAN AND METHODS 9](#_Toc527710119)

[6.1. Planned Analysis 9](#_Toc527710120)

[6.2. Primary Endpoint Analysis: Sample Size Selection 10](#_Toc527710121)

[6.3. Eligibility of Subjects, Exclusions, Missing Data, and Removal of Subjects 10](#_Toc527710122)

[7. ADVERSE EVENT REPORTING 10](#_Toc527710123)

[7.1. Serious Adverse Events 10](#_Toc527710124)

[7.2. Adverse Events 11](#_Toc527710125)

[7.3. Reporting 11](#_Toc527710126)

[8. ADMINISTRATIVE RESPONSIBILITIES 11](#_Toc527710127)

[8.1. Anticipated Study Timeline 11](#_Toc527710128)

[8.2. Criteria for the Termination of the Trial 11](#_Toc527710129)

[8.3. Essential Documentation 11](#_Toc527710130)

[8.4. Subject Confidentiality 12](#_Toc527710131)

[8.5. Quality Control, Quality Assurance, Monitoring 12](#_Toc527710132)

[8.6. Data Integrity 12](#_Toc527710133)

[8.7. Data Handling and Record Keeping 12](#_Toc527710134)

[8.7.1. Study Documentation and Record Keeping 12](#_Toc527710135)

[8.8. Finances 12](#_Toc527710136)

[8.8.1. Study Finance 12](#_Toc527710137)

[8.8.2. Financial Disclosure 13](#_Toc527710138)

# STUDY SYNOPSIS

| Study Title | Evaluating the effect of pulsed near infrared transcranial and intranasal photobiomodulation on real-time EEG signals in older adults at rest |
| --- | --- |
| Study Objective | To evaluate whether pulsed near infrared transcranial and intranasal photobiomodulation produces a reliable effect on neural activity in older adults. |
| Methodology | Double-blind, prospective, crossover |
| Study Duration | Two session evaluation |
| Number of Subjects | Maximum of 20 older adults |
| Major Inclusion/Exclusion Criteria | - 60 years of age or older - No current major psychiatric or neurologic disease - No history of stroke or seizures - All other medical conditions controlled by stable therapy |
| Study Intervention and Planned Use | The experimental intervention will be delivered using the Vielight Neuro Gamma device. The device uses 5 light emitting diodes (810nm wavelength). Four diodes provide near-infrared energy transcranially, while the last does so intranasally, all targeting regions of the Default Mode Network. No significant heat is generated, allowing for a sham device which will be indistinguishable from the intervention to both the subjects and EEG technician. Devices will be utilized concurrently with EEG recording of subject at rest. The devices will be used in a randomized crossover design within the single study visit with the same EEG recordings to be obtained under both sham and active conditions. |
| Study Procedures | 1. Informed Consent 2. Eligibility Assessment 3. First EEG Recording 4. One week washout period 5. Second EEG Recording |
| Primary Endpoint | Change from baseline in overall power spectral density between active and sham session |
| Statistical Methodology | Repeated measures ANOVA with treatment as within subject measure |

# INTRODUCTION

## Study Rationale & Background

Photobiomodulation (PBM) involves the use of low levels of red or near-infrared (NIR) light to either stimulate or inhibit biological cells and tissues via a photochemical mechanism. Transcranial PBM (tPBM) is used to target the delivery of light energy to the brain. It is associated with increased cerebral blood flow, oxygen availability and consumption, adenoside triphophosphate (ATP) production, and improved mitochondrial activity. However, there are questions as to whether near infrared light is capable of penetrating the skull and/or nasal mucosa to reach and produce reliable effects in the brain. The aim of current study is to utilize electroencephalography (EEG) as a method to measure, in real time, the effects of pulsed near infrared transcranial and intranasal photobiomodulation on neural activity.

## Study Device: Vielight Neuro Gamma

The Vielight Neuro Gamma device is a portable, wearable, low-level light delivery device that administers near-infrared light to the brain trans-cranially and intra-nasally and is intended to enhance mental acuity as part of general wellness. The device, shown below, consists of a controller (1), a Nasal Applicator (2), and a Head Set (3).


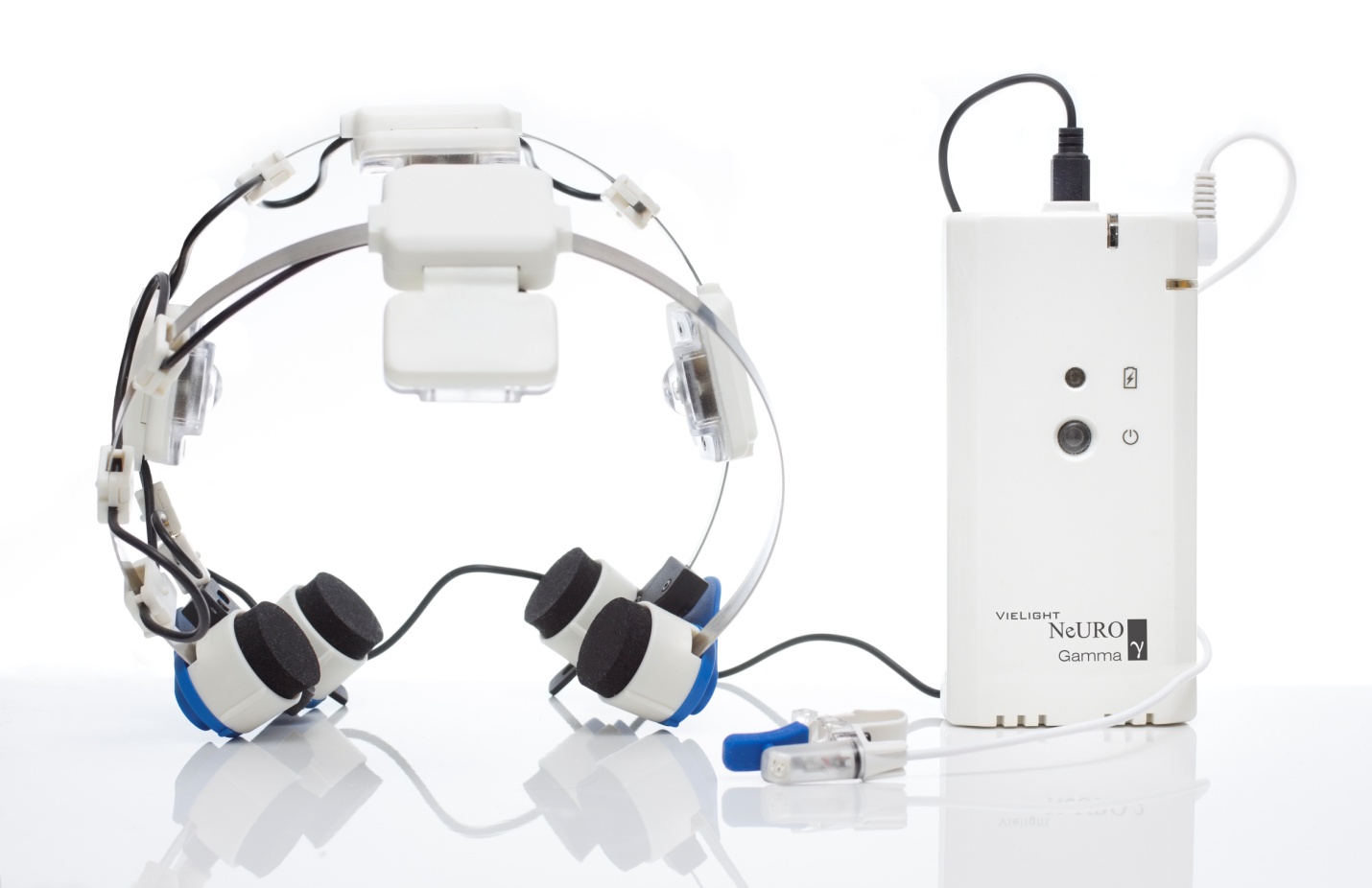


Figure 1. Vielight Neuro Gamma device

The Vielight Neuro Gamma delivers painless, non-invasive, non-thermal, non-laser, pulsed (40 Hz; 50% duty cycle), near-infrared light (810 nm wavelength) through 5 non-laser light emitting diodes (LEDs) over a 20 minute session. The device is powered by three rechargeable NiMH batteries. As shown below, one of the Vielight Neuro Gamma LEDs is placed inside the nose for intranasal transmission of light energy and the remaining four LEDs are positioned over the skull for transcranial transmission.

| 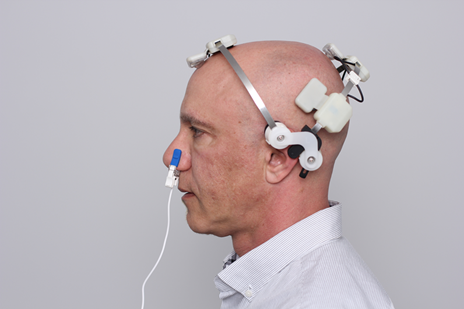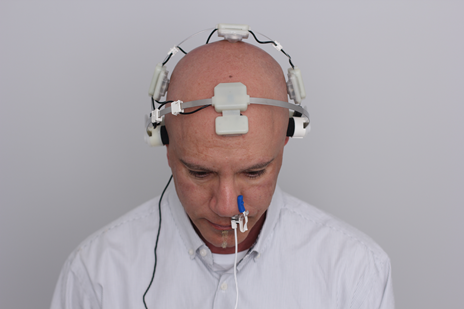  Figure 2. Placement of Vielight Neuro Gamma device |
| --- |

##

## Study Objective

In order to effectively test the capability of pulsed near infrared transcranial and intranasal photobiomodulation to affect neural activity, EEGs using an active device must be compared to sham device EEGs in 20 subjects. This will allow us to reasonably calculate whether there is a significant difference in power spectral density as well as examine other relevant outcomes post-hoc.

## Study Design

This is a non-randomized prospective experimental study.

Study Procedures are as follows:

1. Informed Consent
2. Eligibility Assessment
3. First EEG Recording
4. One week washout period
5. Second EEG Recording

## Study Endpoints

Detailed information about endpoint calculation is provided in Section 6 Statistical Plan and Methods.

## Study Site

This study will be conducted in the offices of Vielight Inc. at 346A Jarvis Street in the downtown Toronto region. This site has been setup to meet the requirements of the EEG system.

# SUBJECT ELIGIBILITY

## Inclusion Criteria

1. Present valid government identification demonstrating age of 60 years or older
2. Capable of speaking English in order to comply with study procedures
3. Capable of reading English in order to complete Informed Consent form and online screening survey

## Exclusion Criteria

1. Unable/unwilling to sign Informed Consent form
2. Mini Mental State Examination (MMSE) score of <27
3. Current major psychiatric or neurologic disease
4. History of stroke or seizures
5. Medical conditions uncontrolled by stable therapy

# STUDY EXECUTION

## Subject Recruitment

Subjects will be recruited from the Greater Toronto Area through recruitment advertisements placed online on sites such as Craigslist or Kijiji and via social media sites including Facebook. Advertisements in the form of posters in the local area surrounding the study site or in newspapers such as NOW! or Eye Weekly may also be used to recruit prospective subjects. Any advertisements will be approved by an Institutional Review Board. A total of 20 subjects are anticipated to complete this study.

## Preliminary Eligibility Assessment

Eligibility screening will be conducted through an online screening form providing a short description of the study and requesting non-medical information. Questions will be related to the inclusion criteria in section 3 above as well as with regards to their ability to comply with study procedures (eg. willingness to remove any religious clothing or wigs to accommodate the EEG cap).

## Study Visit Procedures

**FIRST STUDY VISIT**

A) Study description and Informed Consent

- Study staff will explain the purpose of the study to the participant and what study participation will involve.
- Information must be given in both oral and written form.
- Subjects will be provided a copy of the *Informed Consent Form,* and given as much time as necessary to make an informed decision regarding participation.
- The subject information advises the participant that they are free to refuse their participation in or withdraw from the study at any time.
- The subject will be encouraged to ask questions.
- The investigator or other study staff will then obtain and document informed consent from the participant.
- The subject will be given copies of the signed *Informed Consent Form*.
- If consent is declined, the subject's reason for declining (if provided) will be recorded in the screening log and CRF

B) Assessment of eligibility criteria

- Following consent, study staff will reassess eligibility captured during online screening and record these on the study Case Report Form (CRF).
- Study staff will also confirm the following exclusion criteria and record these on the study Case Report Form (CRF):
  1. No current major psychiatric disorder or neurologic disease
  2. No history of stroke or seizure
  3. No other medical conditions uncontrolled by stable therapy
- Study staff will then conduct the MMSE to determine if the participants score is above 27 (ie. cognitively healthy) and record this on the study Case Report Form (CRF).
- INCIDENTAL FINDINGS: If the subject has a MMSE score of 23 or lower, they will be informed that their score indicates potential cognitive impairment; however, this finding may be erroneous. Subjects will be encouraged to visit a physician in order to be properly assessed for cognitive impairment.
- If the subject does not satisfy eligibility criteria, the subject will not be enrolled and the CRF will indicate the reason for exclusion. A screening log will be maintained listing all subjects screened for the study and will document the reasons for non-participation of non-enrolled subjects.

C) Acquisition of EEG recordings

- Subject will be asked to sit in a comfortable chair, with their back supported and their legs and ankles uncrossed with feet flat on the floor
- Study staff will record any relevant subject details (physical characteristics and demographics) in the Case Report Form. These include but are not limited to: the Subject’s date of birth (YYYY-MM-DD), sex, weight, height, etc.
- Study staff will place the saline-rinsed EEG electrode cap on the subject and may apply additional saline to individual electrode locations in order to ensure good conductivity
- Once the electrode cap is producing reliable readings, the subject will be asked to remain still and quiet with their eyes closed for an initial 10 min Rest-EEG recording
- At the completion of the 10 min recording, study staff will then place the Vielight Neuro Gamma device or Sham device on the subject, as shown in Figure 2 above.
- Study staff may then re-apply additional saline to individual electrode locations in order to ensure good conductivity
- Once the electrode cap is producing reliable readings, the subject will be asked again to remain still and quiet with their eyes closed for a 20 min Rest-EEG recording
- At the completion of the 20 min recording, study staff will then remove the Vielight Neuro Gamma device or Sham device and again re-apply additional saline to individual electrode locations in order to ensure good conductivity
- Once the electrode cap is producing reliable readings, the subject will be asked to remain still and quiet with their eyes closed for a final 10 min Rest-EEG recording

**SECOND STUDY VISIT**

The second study visit will be a replication of Part C of the first visit. At the conclusion of the visit, the subject will be provided financial compensation of $100 CAD for the study.

# STUDY VISIT EQUIPMENT

## Devices and Procedural Purposes

| **Device** | **Purpose** |
| --- | --- |
| Vielight Neuro Gamma | Device being evaluated for effect on neural activity |
| BrainMaster Discovery 20 System | Quantitative EEG system with recording electrode cap |

## Device Safety

Vielight Neuro Gamma: The controller houses three 1.5V NiMH batteries (1900 mAh each) and the controlling electronics for the Neuro Gamma. Both the Headset and Nasal Applicator are connected to the controller with connectors that prevent them from being incorrectly inserted. A single button initiates a software controlled 20-minute therapy – automatically timed but can be discontinued by pressing the button a second time. The maximum power output density to the nasal mucosa is 25 mW/cm^2^ with a 50% duty cycle (total energy delivered per session = 15 J). The maximum power output density to the scalp is 100 mW/cm^2^ with a 50% duty cycle for the posterior band LEDs and 100 mW/cm^2^ with a 50% duty cycle for the anterior band LED (total energy delivered per session = 225 J). All LEDs are well under the exposure limits established by IEC 62471:2006 - *Photobiological safety of lamps and lamp systems*..

BrainMaster Discovery 20 System: The BrainMaster Device does not produce any electro-magnetic fields and does not interfere with the operability of other devices The unit is entirely powered by the USB interface, eliminating the need for batteries, while maintaining subject safety.

# STATISTICAL PLAN AND METHODS

## Planned Analysis

The primary endpoint for this study will be the change from baseline in overall power spectral density between active and sham session. To statistically determine a significant difference a repeated measures ANOVA with treatment as the within-subject measure will be utilized.

Additional exploratory analysis of a variety of measures obtained from the EEG recording will be conducted post-hoc.

## Primary Endpoint Analysis: Sample Size Selection

The sample size of the study was chosen based on a review of the literature for exploratory EEG studies of novel neurotechnologies. Utilizing a sample size of 20 for this initial study will allow us to best estimate effects sizes of critical measures to be examined in follow-up confirmatory studies.

## Eligibility of Subjects, Exclusions, Missing Data, and Removal of Subjects

All subjects responding to the advertisements are potential study candidates and will be screened for eligibility. Every effort will be made to ensure eligibility of the subjects prior to study enrollment. Subjects who do not meet all inclusion/exclusion criteria will not be enrolled or followed in the study. Subjects for whom no data was acquired due to technical failures or those that withdraw consent will be excluded from analysis. Subjects for whom only partial data was acquired will be included in all possible analysis.

# ADVERSE EVENT REPORTING

## Serious Adverse Events

A serious adverse event is any adverse event which occurs during the study visit

i. is life threatening; or

ii. results in permanent impairment of a body function or permanent damage to a body structure; or

iii. necessitates significant intervention, such as major surgery, to prevent permanent impairment of a body function or permanent damage to a body structure; or

iv. requires hospitalization or an extended hospital stay; or

v. results in moderate transient impairment of a body function or transient damage to a body structure; or

vi. requires intervention, such as medication or cardioversion to prevent permanent impairment of a body function or damage to a body structure.

Examples of serious adverse events include death, cardiac tamponade, myocardial infarction, stroke, and seizure.

## Adverse Events

An adverse event is any adverse event that results in minimal transient impairment of a body function or damage to a body structure or which does not require any intervention other than monitoring. Examples of an adverse event include skin reaction or headache at the locations where the Vielight Neuro Gamma device makes contact with the subject's skin.

A pre-existing condition should not be reported as an adverse event unless there has been a substantial change in severity that cannot be attributed to natural history.

## Reporting

As this is a minimal-risk study, no serious adverse events are anticipated. However, any adverse events reported by subjects or observed by the study staff, during the course of the study visit procedures, will be recorded in the subjects CRF along with a brief description and any action taken. All adverse events determined by the principal investigator to be a potential result of study visit procedures, will be reported to the Institutional Review Board and the study sponsor.

# ADMINISTRATIVE RESPONSIBILITIES

## Anticipated Study Timeline

| Recruitment Initiation | D0 |
| --- | --- |
| 20 Subjects Recruited | D0 + 4 weeks |
| Interim Analysis Completed | D0 + 4 weeks +10 days |
| Study Complete | D0 + 6 weeks |

## Criteria for the Termination of the Trial

The study will be terminated upon completion of the recruitment target (N=20) and verification of the data by the study sponsor. The study may also be terminated earlier at the discretion of the sponsor.

## Essential Documentation

The Principal Investigator and other appropriate study personnel will sign the protocol to document their willingness to adhere to this protocol and to conduct the study in accordance with local legal requirements and the International Conference on Harmonization (ICH) guidelines for GCP.

In order to be authorized to initiate the study, the Principal Investigator will ensure the following documentation is obtained:

1. A copy of the dated, documented approval of the IRB to the protocol and any amendments, subject consent forms, advertisements for subject recruitment , subject compensation, and any other documents submitted. The IRB approval should quote version numbers and/or dates of documents reviewed
2. Evidence that the IRB operated in compliance with 21CFR56 (e.g., list of members and affiliations)
3. The Principal Investigator’s current *curriculum vitae* (CV)
4. A copy of the protocol signed with a statement of interest by the Principal Investigator

All other relevant documents essential prior to study initiation should be placed appropriately in the site’s study master files.

## Subject Confidentiality

The confidentiality of subjects enrolled in this study will be maintained at all times.

All study reports and communications relating to subjects will identify each subject by their subject number and initials only. Investigators will maintain a log enabling them to identify clearly each subject enrolled.

## Quality Control, Quality Assurance, Monitoring

The data from source documents will be entered into CRFs. The data will be subjected to manual review for completeness, logic, accuracy, and consistency. A final monitoring report will be generated demonstrating compliance to the protocol.

## Data Integrity

Data integrity is ensured through monitoring by the sponsor of source document to CRF verification.

## Data Handling and Record Keeping

### Study Documentation and Record Keeping

The study sponsor will retain all documents associated with the study for at least two years. Study documents shall be retained for a longer period, if required by the applicable regulatory authority or by agreement with the sponsor.

## Finances

### Study Finance

Subjects will be provided $100 CAD as compensation for their involvement at the end of the second study visit, following the completion of all study procedures.

### Financial Disclosure

The investigator must provide the sponsor with sufficient and accurate financial information to meet regulatory disclosure requirements (21 CFR 54). The investigator will update this information if any relevant changes occur during the investigation and for one year following the completion of the study.
